# Prevalence of handcrafted milk tea consumption among Chinese college students and associations with unhealthy lifestyles and sensation seeking

**DOI:** 10.3389/fpsyg.2026.1800521

**Published:** 2026-03-20

**Authors:** Zaidong Hu, Chunmei Hu

**Affiliations:** Mental Health Education and Counseling Center, Chongqing University of Arts and Sciences, Chongqing, China

**Keywords:** association, college students, handcrafted milk tea consumption, sensation seeking, unhealthy lifestyle

## Abstract

**Background:**

College students are the main consumer group of handcrafted milk tea. Excessive consumption of handcrafted milk tea may harm their physical and mental health and even lead to milk tea addiction. Despite growing consumption, relatively few studies have examined handcrafted milk tea consumption and its associated factors among college students. This study aimed to explore the prevalence of handcrafted milk tea consumption among Chinese college students and its associations with unhealthy lifestyle and sensation seeking, thereby providing empirical evidence for health education related to milk tea consumption.

**Methods:**

Data were collected from 3,596 college students using a cross-sectional survey design, in which participants completed questionnaires assessing handcrafted milk tea consumption, unhealthy lifestyle, and sensation seeking. Statistical analyses were performed using SPSS 23.0, mainly including descriptive statistics, chi-square tests, and multivariable logistic regression analyses.

**Results:**

(1) The prevalence of handcrafted milk tea consumption among college students was 75.2%, and the excessive consumption rate was 7.0%. The average daily intake was 111.78 mL, and the average daily expenditure was 3.16 yuan. (2) Regarding unhealthy lifestyle factors, carbonated beverage consumption (OR of occasionally consumption:2.425, OR of frequently consumption:3.976), dessert intake (OR:1.761), fried food consumption (OR of less frequently consumption:1.734, OR of frequently consumption:1.827), insufficient physical activity (OR:1.389), and alcohol consumption (OR:1.551) were positively associated with handcrafted milk tea consumption. Students engaging in these unhealthy lifestyle behaviors had an increased prevalence of handcrafted milk tea consumption. (3) High sensation seeking levels (OR: 1.861) were positively associated with handcrafted milk tea consumption among college students. Students with higher levels of sensation seeking had an increased prevalence of handcrafted milk tea consumption.

**Conclusion:**

More than 70% of college students consumed handcrafted milk tea, and the rate of excessive consumption appeared to be increasing. Handcrafted milk tea consumption among college students tended to cluster with multiple unhealthy lifestyle behaviors. College students with high sensation seeking were more likely to consume handcrafted milk tea. Universities should implement targeted educational initiatives based on the identified associated factors to guide students toward rational handcrafted milk tea consumption.

## 1 Introduction

Handcrafted milk tea refers to a broad category of beverages that are freshly prepared and sold in specialty drink shops. It primarily includes formulated drinks based on tea extract concentrates, such as milk tea, fruit tea, cheese tea, and freshly brewed coffee (Huang et al., 2021).

In the early 20th century, milk tea entered the Chinese market and subsequently experienced rapid growth, gradually emerging as a popular form of leisure consumption among young people. Initially, milk tea was primarily prepared by dissolving powdered milk tea in hot water. As consumers’ purchasing power increased and their demands for taste and product diversity changed, milk tea progressively evolved into freshly prepared, ready-to-drink tea beverages (Wingnam et al., 2021). At present, milk tea shops in China primarily adopt a product mix strategy centered on “milk tea plus other tea-based beverages.” In contrast to powdered milk tea, handcrafted milk tea incorporates fresh tea leaves and diverse ingredients, including milk, fruits, pearls, and coconut jelly, thereby better satisfy consumers’ expectations regarding perceived healthfulness and taste and attracting a growing consumer base. Consequently, handcrafted milk tea consumption has emerged as a fashionable and widely adopted leisure practice. Along with rising consumption, the scale of handcrafted milk tea shops has continued to expand, with the total number of milk tea outlets in China approaching 500,000 nationwide by 2022. Handcrafted milk tea shops place strong emphasis on aesthetic and fashionable store design, while continuously innovating product varieties and flavors. By leveraging social media for word-of-mouth promotion, the handcrafted milk tea industry has further expanded its market reach. Overall, the handcrafted milk tea industry has entered a stage of brand-oriented development, giving rise to numerous well-known handcrafted milk tea brands (Qianyi, 2024).

As a representative type of novel sugar-sweetened beverages, handcrafted milk tea is characterized by nutritional imbalance, high energy density, elevated caffeine content, high levels of added sugar and fat, and low protein content. The caloric content of handcrafted milk tea has been reported to exceed that of traditional pre-packaged sugar-sweetened beverages of comparable size (Shi et al., 2023). Excessive consumption of handcrafted milk tea may be with adverse physical and mental health outcomes.

College students represent a key population of consumers of handcrafted milk tea (Wingnam et al., 2021). Ongoing product innovation in the handcrafted milk tea industry, together with the promotion of a fashionable trendy handcrafted milk tea consumption culture on social media, has attracted college students to continue consumption. As the main consumer group of handcrafted milk tea, drinking handcrafted milk tea may pose a threat to the physical and mental health of college students. Therefore, it is necessary to investigate the prevalence of handcrafted milk tea consumption and its associated factors among college students. The research results can provide a reference for universities to design and implement educational initiatives promoting rational handcrafted milk tea consumption.

### 1.1 Negative effect of handcrafted milk tea consumption to college students’ physical and mental health

The handcrafted milk tea consumption is related to the physical and mental health of college students. Previous studies suggest that excessive handcrafted milk tea consumption can pose a threat to college students’ health.

First, high levels of handcrafted milk tea consumption may adversely affect college students’ physical health. For example, excessive intake has been associated with elevated risks of overweight and obesity, hypertension, and diabetes among college students (Li et al., 2024a,b; Gu et al., 2024). Among male college students, greater handcrafted milk tea consumption wad associated higher heart rate variability, whereas this associations were not observed among female students (Chai et al., 2022).

Second, excessive handcrafted milk tea consumption may exacerbate negative psychological symptoms among college students. This association may be partly attributable to the excessive intake of sugar, caffeine, and fat resulting from high levels of handcrafted milk tea consumption (Jahrami et al., 2020; Liu et al., 2019), thereby elevating the risk of stress dysregulation, anxiety and irritability, depressive symptoms, and suicidal ideation (Wu et al., 2022; Gu et al., 2024; Qu et al., 2023; Li et al., 2024a,b).

Third, handcrafted milk tea consumption may play a potential role in emotion regulation among college students. When experiencing negative emotions, college students may consume handcrafted milk tea as a means of alleviating emotional distress. Nevertheless, the appealing taste and affordability of handcrafted milk tea render it a readily accessible emotion-regulation strategy, which may reinforce habitual reliance on handcrafted milk tea and increase the risk of addiction. Dependence on handcrafted milk tea may impede the development of adaptive emotion regulation strategies, thereby further intensify negative emotions and contribute to depressive symptoms, anxiety, and suicidal ideation (Qu et al., 2023).

Given the numerous potential harms associated with excessive handcrafted milk tea consumption among college students, this study aims to examine the prevalence and associated factors of handcrafted milk tea consumption. These findings may offer empirical support for universities in developing targeted educational interventions regarding handcrafted milk tea consumption.

### 1.2 Association between handcrafted milk tea consumption and unhealthy lifestyle behaviors among college students

Lifestyle represents a comprehensive manifestation of behavioral patterns and daily habits gradually formed during individual development, including dietary habits, levels of physical activity, smoking, and alcohol consumption (Silva et al., 2020). When individuals exhibit one form of unhealthy lifestyle behavior, they are more likely to engage in additional unhealthy lifestyle behaviors (Wang et al., 2023).

Handcrafted milk tea consumption is one of the prevalent unhealthy dietary habits among college students. As handcrafted milk tea is a relatively new type of sugar-sweetened beverage, research examining its association with unhealthy lifestyle behaviors remains limited. Whereas numerous studies have investigated the association between traditional sugar-sweetened beverage consumption and unhealthy lifestyles among college students. Previous studies have demonstrated that unhealthy lifestyle behaviors, including physical inactivity, smoking, alcohol use, and poor dietary practices, were positively associated with the sugar-sweetened beverages consumption among college students in different countries (Audrey et al., 2018). Hu et al. (2019a,b) reported that smoking and alcohol consumption were significantly associated with energy drink consumption among Chinese college students, with higher consumption rates observed among students who smoked or drank alcohol. Phulkerd et al. (2022) found that insufficient physical activity and smoking were associated with higher levels of sugar-sweetened beverage consumption among Thai college students, with physically inactive or smoking students consuming larger amounts. High fast-food intake had been identified as being associated with elevated sugar-sweetened beverage consumption among Malaysian college students, as fast-food outlets frequently bundled carbonated beverages with fried foods and desserts, leading fast-food consumers to ingest greater amounts of these foods (Norliza et al., 2019).

Based on prior findings, this study investigates the association between handcrafted milk tea consumption and unhealthy lifestyle behaviors among college students. In this study, unhealthy lifestyle behaviors primarily include insufficient physical activity, smoking, alcohol consumption, and unhealthy dietary behaviors. The unhealthy dietary behaviors specifically referred to the consumption of fried foods, desserts, and sugar-sweetened beverages. Given that carbonated beverages represent the most widely consumed sugar-sweetened beverages among adolescents in Europe, North America, Oceania, and China (Hong et al., 2017; Liu et al., 2020), carbonated beverages were therefore selected for assessment of sugar-sweetened beverage consumption in this study.

### 1.3 Association between handcrafted milk tea consumption and sensation seeking among college students

Sensation seeking is defined as a personality trait characterized by individuals’ tendency to pursue novel, intense, varied, and complex sensory experiences, and to engage in behaviors to achieve these experiences. It is associated with adolescents’ engagement in risk behaviors, such as smoking, alcohol consumption, and unhealthy eating (Duell and Steinberg, 2020; Hu et al., 2019a,b). Currently, few studies have investigated the association between handcrafted milk tea consumption and sensation seeking.

Previous studies have examined the association between energy drink consumption and sensation seeking among college students, indicating that sensation seeking was positively associated with energy drink consumption. Since energy drink marketing strategies often portray consumption as fashionable, trendy, and stimulating, which aligns with college students’ sensation seeking tendencies; consequently, individuals with higher sensation seeking are more likely to consume energy drinks (Hu et al., 2019a,b). In China, the handcrafted milk tea industry has leveraged social media to position handcrafted milk tea consumption as a trendy and fashionable form of leisure. Additionally, major handcrafted milk tea brands emphasize marketing innovation, continuously introducing novel store designs, beverage flavors, packaging, and consumer experiences to attract young consumers and boost sales. Based on these observations, we propose that sensation seeking among college students may be related to their handcrafted milk tea intake, which this study seeks to investigate.

In summary, this study aims to examine the consumption patterns of handcrafted milk tea among Chinese college students and to explore the relationships between milk tea consumption, unhealthy lifestyle behaviors, and sensation seeking. The results of this research may offer valuable insights for designing health education interventions aimed at promoting more rational handcrafted milk tea consumption among college students.

## 2 Materials and methods

### 2.1 Study design

This study used a cross-sectional research design. It is a descriptive study that involves analyzing the information of a certain population at a specific point in time and can also be used to determine the relationships between variables. This method may limit its universality due to potential sampling bias, as the participants were not randomly selected from a broader group of college students.

### 2.2 Setting and participants

In this study, we used convenience sampling method to recruit college students from three universities in Chongqing, China. The investigators had been conducting teaching and research projects for these three universities for a long time. We had good relationships with many counselors and teachers, and they can assist in distributing the questionnaires. The sample size was determined based on the principles of multivariate regression analysis, which requires that each independent variable should have at least 10–15 participants. This study included 11 potential predictors in the regression model, thus requiring at least 110–165 participants. Our final sample of 3,596 exceeded this minimum requirement, providing sufficient statistical power to detect meaningful associations. The inclusion criteria for participants were as follows: (1) age ≥18 years; (2) studying in school from March to April in 2025; (3) voluntarily signing the “Informed Consent Form for the Survey.” The exclusion criteria included unwillingness to sign the “Informed Consent Form for the Survey” and refusal to participate in the survey.

Initially, 3,900 college students met the inclusion criteria and voluntarily completed the questionnaire. Due to the incomplete or repetitive responses in the questionnaires of 304 college students, their questionnaires were treated as invalid and were not included in the statistical analysis. The final sample was 3,596, yielding an effective response rate of 92.21%. Among the participants, 1,173 were male (32.6%) and 2,423 were female (67.4%); 2,131 students (59.3%) majored in liberal arts, and 1,465 students (40.7%) majored in sciences; 2,165 were freshmen (60.2%), 941 were sophomores (26.2%), and 490 were juniors (13.6%); 1,284 were from urban areas (35.7%), and 2,312 were from rural areas (64.3%). The mean age of the participants was 19.72 years (SD = 5.91).

### 2.3 Data collection

Data collection was carried out from March to April in 2025. First, ethical approval was obtained from the Ethics Committee of the participating university (Approval Number: CQWLDF0030). Second, professional survey staff contacted student advisors to inform them of the survey content and objectives, and to obtain their consent and support, as well as to determine the time and location for data collection. Third, during formal data collection, student advisors organized participants to complete paper questionnaires in classrooms. Survey staff explained the study’s purpose and procedures on-site, emphasized anonymity and confidentiality, and distributed informed consent forms; questionnaires were then provided to students who signed the consent forms, completed on the spot, and immediately collected. Fourth, survey staff checked and organized the returned questionnaires, excluded incomplete or patterned responses as invalid, and completed data entry and analysis.

### 2.4 Measurement tools

#### 2.4.1 Handcrafted milk tea consumption questionnaire

Drawing on questionnaires employed by Li et al. (2024a,b), a survey with three items was constructed to assess college students’ amount, cost, and frequency of handcrafted milk tea consumption during the past week. Initially, handcrafted milk tea was clearly defined in the questionnaire as freshly made beverages purchased from milk tea stores to facilitate accurate self-reporting by participants. Subsequently, respondents completed the following three questions. (1) “During the past week, what was your total volume of handcrafted milk tea consumed from milk tea shops (in milliliters)?” Participants estimated their intake based on typical market sizes, including small (~350 mL), medium (~500 mL), large (~700 mL), and bucket-sized (~1,000 mL) servings. (2) “How much money (in RMB) did you spend on handcrafted milk tea during the past week?” Participants reported their actual expenditure. (3) “How many occasions did you consume handcrafted milk tea from milk tea stores during the past week, with one occasion defined as finishing at least one cup?” The response categories were “0 times weekly,” “once weekly,” “2–3 times weekly,” “4–6 times weekly,” “once daily,” “twice daily,” and “three or more times daily.” Based on participants’ responses, consumption was categorized as non-consumption (0 times), normal consumption (1–3 times weekly), and excessive consumption (4 times or more weekly). Due to the limited number of participants reporting excessive consumption, normal and excessive consumers were combined into “consumption group.”

#### 2.4.2 Unhealthy lifestyle questionnaire

This questionnaire assessed college students’ unhealthy diet behaviors, physical activity, smoking, and alcohol consumption. The items were adapted from the Comprehensive Questionnaire on College Students’ Risk Behaviors developed by Ji (2007). This questionnaire has been widely used in studies on risk behaviors among Chinese adolescents (Hu et al., 2019a,b).

(1) Unhealthy dietary behaviors were measured by investigating college students’ consumption frequency of carbonated drinks, desserts, and fried foods in the previous week.

First, carbonated beverage consumption was assessed using the item “How many times per day did you usually drink carbonated beverages during the past week?” The response categories included “0 times,” “1 time,” “2 times,” up to “7 times or more daily.” Based on responses, participants were categorized into three groups: non-consumption, occasional consumption (one to two times daily), and frequent consumption (at least three times daily).

Second, dessert consumption was assessed using the item “How many times did you eat desserts during the past week?” The response categories included “0 times,” “1–6 times,” “once daily,” and “twice or more daily.” Participants were categorized into three groups: non-consumption, occasional consumption (one to six times weekly), and frequent consumption (one or more times daily). In this study, only a small proportion of participants reported frequent dessert consumption. Thus, participants who occasionally or frequently consumed desserts were merged into “dessert consumption” in the analyses.

Third, fried food consumption was assessed using the item “How many times did you eat fried foods during the past week?” The response categories included “0 times,” “1–6 times,” and “7 times or more.” Participants were categorized into three groups: non-consumption, low consumption (one to two times weekly), and frequent consumption (at least three times weekly).

(2) Physical activity was assessed using the item “How many days did you participate in at least 60 min of physical activity each day during the past week?” Participants who reported two or fewer days per week with at least 60 min of activity were classified as having insufficient physical activity.

(3) Smoking was assessed using the item “Have you smoked cigarettes during the past 30 days?” The response categories were “no,” “once,” and “more than once.” Given the small proportion of participants reporting smoking more than once, the categories “once” and “more than once” were merged into “smoking” group for analysis.

(4) Alcohol consumption was assessed using the item “Have you consumed alcohol during the past 30 days?” The response categories were “no,” “once,” and “more than once.” Given the small proportion of participants reporting drinking more than once, the categories “once” and “more than once” were merged into “drinking” group for analysis.

#### 2.4.3 Sensation seeking scale

This study employed the Sensation Seeking Scale originally developed by Zuckerman et al. (1993) and later revised by Hu et al. (2019a). This questionnaire has been widely used in studies among Chinese adolescents (Hu et al., 2019a,b). The scale consisted of 11 items. Items were scored on a dichotomous scale, with “yes” scored as 1 and “no” scored as 0. Participants were classified into group based on their total scores (Lee et al., 2016; Hu et al., 2019a; Hu and Wang, 2019). Participants with total scores higher than 6 were categorized as the high sensation seeking group. Those with total scores of 6 or below were classified as the low sensation seeking group. In this study, the internal consistency coefficient (Cronbach’s *α*) of the scale was 0.71.

### 2.5 Covariates variables

According to previous research, there were significant differences in sugar-sweetened beverages (such as milk tea and energy drinks) consumption rate among college students with different gender, grade, major and origin (Li et al., 2024b; Gu et al., 2024; Hu et al., 2019a,b). Therefore, in this study, gender, major, grade and origin were taken as covariates variables.

### 2.6 Statistical analysis

SPSS 23.0 was used for data entry and statistical analyses. Counting data were described using frequencies and percentages, while measurement data that follow a normal distribution were presented as mean ± standard deviation. Descriptive statistics were conducted to describe the prevalence of handcrafted milk tea consumption among college students. Chi-square tests were used to examine differences in handcrafted milk tea consumption rates across students with different characteristics. When the variable classification is greater than or equal to 3, if the chi-square test result showed statistical significance, Bonferroni correction was used for multiple comparisons to examine the differences between groups. Multivariable logistic regression analyses were conducted to investigate the associations between handcrafted milk tea consumption and unhealthy lifestyles and sensation seeking with unadjusted and adjusted for covariates to test the stability of the results. Variables that showed significant differences in handcrafted milk tea consumption rates in the chi-square analyses were included as independent variables. Collinearity among independent variables was evaluated by checking the variance inflation factor (VIF) and tolerance; when VIF < 5 and tolerance >0.2, it indicated no substantial multicollinearity (Field, 2009). A confidence interval of 95% and a margin of error of 0.05 were considered statistically significant throughout the analysis.

## 3 Results

### 3.1 Examination of common method bias

Since all the data in this study were obtained from the self-reports of the participants, the Harman single-factor test was conducted prior to data analysis to examine for common method bias. The results showed that there were seven factors with eigenvalues >1, and the first factor explained 19.12% of the variance, less than the critical criterion of 40% (Zhou and Long, 2004), so there was no significant common method bias.

### 3.2 Handcrafted milk tea consumption among college students

Descriptive statistics showed that 2,703 of the 3,596 college students reported consuming handcrafted milk tea, accounting for 75.2% of the sample. Among these consumers, 2,453 students (68.2%) were normal consumers, and 250 students (7.0%) were excessive consumers (Table 1). The mean daily intake of handcrafted milk tea was 111.78 mL (SD = 136.33). The mean daily expenditure on handcrafted milk tea was 3.16 CNY (SD = 4.06).

**Table 1:** Handcrafted milk tea consumption rates among college students (*n* = 3,596).

Handcrafted milk tea consumption rates		*N* (%)
No consumption	0 times weekly	893 (24.8)
Normal consumption (1–3 times weekly)	once weekly	1,509 (42.0)
	2–3 times weekly	944 (26.2)
Excessive consumption (4 times or more weekly)	4–6 times weekly	155 (4.3)
	once daily	39 (1.1)
	2 times daily	32 (0.9)
	3 times or more daily	24 (0.7)
Total		3,596 (100)

*N* (%) represents the number of participants (percentage), and the same applies hereafter.

### 3.3 Differences in handcrafted milk tea consumption rates among college students with different characteristics

Chi-square test results (Table 2) indicated that handcrafted milk tea consumption rates differed significantly by gender, academic major, grade level, carbonated beverage consumption, dessert intake, fried food intake, physical activity level, alcohol consumption, and sensation seeking. The consumption rate among female students (82.0%) was higher than that among male students (61.1%) (*χ^2^* = 183.880, *p* < 0.001). Arts students showed a higher consumption rate (78.9%) than sciences students (69.8%) (*χ^2^* = 71.168, *p* = <0.001). Freshmen had a higher consumption rate (80.0%) than sophomores and juniors (69.3 and 65.1%, respectively) (*χ^2^* = 71.108, *p* < 0.001, *p′* < 0.001), while there was no significant difference in consumption rate between sophomores and juniors. Students who frequently consumed carbonated beverages had higher consumption rates (84.9%) than those who occasionally consumed carbonated beverages (79.7%), and those who occasionally or frequently consumed carbonated beverages had higher consumption rates (79.7 and 84.9%) than those who did not consume carbonated beverages (61.4%) (*χ^2^* = 174.095, *p* < 0.001). Students who consumed desserts had higher consumption rate (82.1%) than those who did not consume desserts (60.1%) (*χ^2^* = 201.646, *p* < 0.001). Students who consumed fried foods less frequently or frequently had higher consumption rates (80.3 and 81.8%) than those who did not consume fried foods (61.8%) (*χ^2^* = 147.793, *p* < 0.001), while there was no significant difference in consumption rate between the groups that consumed less and frequently consumed fried food. Students with insufficient physical activity had higher consumption rate (76.2%) than those with sufficient physical activity (72.5%) (*χ^2^* = 5.390, *p* = 0.020). Students who consumed alcohol had higher consumption rate (78.4%) than those who did not consume alcohol (74.0%) (*χ^2^* = 7.215, *p* = 0.007). Students with high sensation seeking had higher consumption rate (83.7%) than those with low sensation seeking (73.2%) (*χ^2^* = 32.863, *p* = 0.002). No significant differences in handcrafted milk tea consumption rates were observed among students from different origins or smoking status (*χ^2^* = 0.509, *p* = 0.476). No significant differences in handcrafted milk tea consumption rates were observed among college students with different smoking statuses. (*χ^2^* = 0.219, *p* = 0.639).

**Table 2:** Comparison of handcrafted milk tea consumption rates among college students with different characteristics (*n* = 3,596).

Characteristics		*N* (%)	Handcrafted milk tea consumption		*χ^2^*	*p*
			No consumption	Consumption		
Gender	Male	1,173 (32.6)	456 (38.9)	717 (61.1)	183.880	<0.001
	Female	2,423 (67.4)	437 (18.0)	1986 (82.0)		
Academic major	Arts	2,131 (59.3)	450 (21.1)	1,681 (78.9)	71.168	<0.001
	Sciences	1,465 (40.7)	443 (30.2)	1,022 (69.8)		
Grade	Freshman	2,165 (60.2)	433 (20.0)_a_	1732 (80.0)_a_	71.108	<0.001
	Sophomore	941 (26.2)	289 (30.7)_b_	652 (69.3)_b_		
	Junior	490 (13.6)	171 (34.9)_b_	319 (65.1)_b_		
Origin of student	Urban	1,284 (35.7)	310 (24.1)	974 (75.9)	0.509	0.476
	Rural	2,312 (64.3)	583 (25.2)	1729 (74.8)		
Carbonated beverage consumption	No consumption	1,128 (31.4)	435 (38.6)_a_	693 (61.4)_a_	174.095	<0.001
	Occasionally consumption	1,639 (45.6)	333 (20.3)_b_	1,306 (79.7)_b_		
	Frequently consumption	829 (23.1)	125 (15.1)_c_	704 (84.9)_c_		
Dessert consumption	No consumption	1,139 (31.7)	454 (39.9)	685 (60.1)	201.646	<0.001
	Consumption	2,457 (68.3)	439 (17.9)	2018 (82.1)		
Fried foods consumption	No consumption	1,058 (29.4)	404 (38.2)_a_	654 (61.8)_a_	147.793	<0.001
	Less frequently consumption	1781 (49.5)	351 (19.7) _b_	1,430 (80.3) _b_		
	Frequently consumption	757 (21.1)	138 (18.2)_b_	619 (81.8)_b_		
Physical activity	Sufficient	1,034 (28.8)	284 (27.5)	750 (72.5)	5.390	0.020
	Insufficient	2,562 (71.2)	609 (23.8)	1953 (76.2)		
Smoking	No smoking	3,235 (90.0)	807 (24.9)	2,428 (75.1)	0.219	0.639
	Smoking	361 (10.0)	86 (23.8)	275 (76.2)		
Alcohol Consumption	No drinking	2,668 (74.2)	693 (26.0)	1975 (74.0)	7.215	0.007
	Drinking	928 (25.8)	200 (21.6)	728 (78.4)		
Sensation seeking	Low level	2,910 (80.9)	781 (26.8)	2,129 (73.2)	32.863	0.002
	High level	686 (19.1)	112 (16.3)	574 (83.7)		

The categories of grade, carbonated beverages consumption and fried foods consumption were divided into three groups. After the chi-square test results were significant, Bonferroni correction was used for multiple comparisons to examine the differences between different groups. The same subscript letters indicate no statistically significant difference between the groups, while different subscript letters indicate statistically significant differences.

### 3.4 Multivariable logistic regression analysis of the relationships between handcrafted milk tea consumption and unhealthy lifestyle and sensation seeking among college students

Before conducting multivariable logistic regression analysis, a multiple linear regression analysis was used to test whether there was multicollinearity among the independent variables. The result indicated that all VIF values ranged from 1.035 to 1.166 (all <5), and all tolerance levels ranged from 0.792 to 0.966 (all >0.2), indicating no significant multicollinearity. Variables that showed significant differences in the chi-square analyses—gender (male = 1, female = 2), academic major (liberal arts = 1, sciences = 2), grade (freshman = 1, sophomore = 2, junior = 3), carbonated beverage consumption (non-consumption = 0, occasional consumption = 1, frequent consumption = 2), dessert consumption (non-consumption = 0, consumption = 1), fried food consumption (non-consumption = 0, infrequent consumption = 1, frequent consumption = 2), physical activity level (adequate = 0, insufficient = 1), alcohol consumption (non-consumption = 0, consumption = 1), and sensation seeking (low = 0, high = 1)—were included as independent variables, while handcrafted milk tea consumption among college students was included as the dependent variable (non-consumption = 0, consumption = 1) in the multivariable logistic regression model.

The results (Table 3) showed that, after adjusting gender, academic major and grade, unhealthy lifestyle behaviors and high sensation seeking remained significantly positively associated with handcrafted milk tea consumption among college students. The students who consumed carbonated beverages [occasionally consumptions: odds ratio (*OR*) = 2.425, 95%*CI* = 2.001 ~ 2.938, *p* < 0.001; frequently consumption: *OR* = 3.976, 95%*CI* = 3.084 ~ 5.126, *p* < 0.001], ate desserts (*OR* = 1.761, 95%*CI* = 1.468 ~ 2.112, *p* < 0.001), ate fried foods (less frequently consumption: *OR* = 1.734, 95%*CI* = 1.428 ~ 2.107, *p* < 0.001; frequently consumption: *OR* = 1.827, 95%*CI* = 1.418 ~ 2.354, *p* < 0.001), engaged insufficient physical activity (*OR* = 1.389, 95%*CI* = 1.150 ~ 1.677, *p* = 0.001), drank alcohol (*OR* = 1.551, 95%*CI* = 1.258 ~ 1.911, *p* < 0.001), and demonstrated high sensation seeking (*OR* = 1.861, 95%*CI* = 1.457 ~ 2.377, *p* < 0.001) had an increased prevalence of handcrafted milk tea consumption. Hosmer Lemeshow goodness-of-fit test result showed that the chi-square value was 6.124 and *p*-value is 0.633 (>0.5), indicating that the adjusted model had a good fit.

**Table 3:** Logistic regression analysis of the associations between handcrafted milk tea consumption and unhealthy lifestyle behaviors and sensation seeking among college students (*n* = 3,596).

Independent Variables		Reference group	Unadjusted			Adjusted		
			OR (95% CI)	*p*	Beta	OR (95% CI)	*p*	Beta
Carbonated beverage consumption	Occasionally consumption	No consumption	2.046 (1.709 ~ 2.450)	<0.001	0.716	2.425 (2.001 ~ 2.938)	<0.001	0.886
	Frequently consumption		2.903 (2.288 ~ 3.682)	<0.001	1.066	3.976 (3.084 ~ 5.126)	<0.001	1.380
Dessert consumption	Consumption	No consumption	2.324 (1.960 ~ 2.574)	<0.001	0.843	1.761 (1.468 ~ 2.112)	<0.001	0.566
Fried foods consumption	Less frequently consumption	No consumption	1.796 (1.491 ~ 2.164)	<0.001	0.586	1.734 (1.428 ~ 2.107)	<0.001	0.551
	Frequently consumption		1.676 (1.316 ~ 2.133)	<0.001	0.516	1.827 (1.418 ~ 2.354)	<0.001	0.603
Physical activity	Insufficient	Sufficient	1.462 (1.225 ~ 1.746)	<0.001	0.380	1.389 (1.150 ~ 1.677)	0.001	0.329
Alcohol consumption	Drinking	No drinking	1.034 (0.853 ~ 1.254)	0.731	0.034	1.551 (1.258 ~ 1.911)	<0.001	0.439
Sensation seeking	High level	Low level	2.000 (1.583 ~ 2.527)	<0.001	0.693	1.861 (1.457 ~ 2.377)	<0.001	0.621

## 4 Discussion

### 4.1 Basic characteristics of handcrafted milk tea consumption among college students

This study found that the prevalence of handcrafted milk tea consumption among college students was 75.2%, with an excessive consumption rate of 7.0%. Notably, compared with the survey results on Chinese college students’ consumption of handcrafted milk tea by He Qu et al. (2023), the consumption rate was slightly lower than 77%, and the excessive consumption rate was substantially higher than 2.6% reported in a previous survey of college students. Taken together, these findings indicate that handcrafted milk tea consumption is highly prevalent among college students and that excessive consumption shows an increasing trend. This trend may be partly explained by college students’ limited awareness of the ingredients of handcrafted milk tea and their potential health risks. A survey conducted by Zhao et al. (2021) showed that college students had a low level of awareness regarding the added sugar content in handcrafted milk tea and the health risks associated with excessive sugar intake. Importantly, the added sugars and tea polyphenols contained in handcrafted milk tea can stimulate dopamine release in the brain, caffeine can accelerate adrenaline secretion and produce pleasurable sensations and reduce fatigue in consumers; in addition, coupled with insufficient awareness of the potential harms of handcrafted milk tea consumption, these aforementioned factors may lead students to drink handcrafted milk tea at high frequencies and develop handcrafted milk tea dependence (Qu et al., 2023). Based on these findings, universities should strengthen health education concerning the ingredients and potential health risks of handcrafted milk tea. So, universities should guide students toward rational consumption of handcrafted milk tea.

The findings showed that handcrafted milk tea consumption rates were significantly higher among female students, arts majors, freshmen, students who consumed carbonated beverages, desserts, and fried foods, those with insufficient physical activity, those who reported alcohol consumption, and students with higher levels of sensation seeking, compared with their respective counterparts. This result was partially consistent with the findings of Li et al. (2024b) and Gu et al. (2024). Their studies also revealed significant differences in the milk tea consumption rates among college students of different genders, majors, and grades. Furthermore, this study revealed that there were significant differences in the milk tea consumption rates among college students with different unhealthy lifestyles and sensation seeking. Accordingly, when implementing handcrafted milk tea consumption education among college students, differences in consumption characteristics across subgroups should be considered, with enhanced educational efforts specifically targeted toward high-consumption populations.

### 4.2 Associations of handcrafted milk tea consumption with unhealthy lifestyle behaviors and sensation seeking among college students

Multivariable logistic regression analyses indicated that handcrafted milk tea consumption among college students was positively associated with carbonated beverage consumption, dessert intake, fried food intake, insufficient physical activity, alcohol consumption, and high sensation seeking.

First, college students who consumed carbonated beverages occasionally consumptions (occasionally consumptions: *OR* = 2.425, *p* < 0.001; frequently consumption: *OR* = 3.976, *p* < 0.001), ate desserts (*OR* = 1.761, *p* < 0.001) and fried foods (less frequently consumption: *OR* = 1.734, *p* < 0.001; frequently consumption: *OR* = 1.827, *p* < 0.001), engaged in insufficient physical activity (*OR* = 1.389, *p* = 0.001), and reported alcohol consumption (*OR* = 1.551, *p* < 0.001) had an increased prevalence of handcrafted milk tea consumption. This indicated that unhealthy lifestyles behaviors tend to cluster with handcrafted milk tea consumption (Hu et al., 2019b; Guo et al., 2013). This clustering effect may be attributed to lower nutritional knowledge and reduced health consciousness among students with unhealthy lifestyles, which may increase their likelihood of overlooking potential health risks and engaging in multiple unhealthy behaviors (Du et al., 2022; Liu et al., 2017). Therefore, universities should strengthen the cultivation of healthy lifestyles within health education programs, for example by disseminating knowledge about common types, manifestations, risks, and coping strategies of unhealthy lifestyles, and by implementing behavioral training for healthy lifestyle practices.

Second, college students with high sensation seeking had an increased prevalence of handcrafted milk tea consumption (*OR* = 1.861, *p* < 0.001), which may be related to the innovative nature and personalized marketing strategies of handcrafted milk tea products (Wingnam et al., 2021). The handcrafted milk tea industry places strong emphasis on product innovation and brand marketing, continuously introducing new product varieties, flavors, and ingredients, which effectively caters to young people’s psychological needs for novelty, fashion and trendiness. Meanwhile, handcrafted milk tea companies and retailers focus on shaping personalized brand identities through customized store designs, visually appealing beverages, and packaging, and construct handcrafted milk tea consumption as a fashionable social culture via online posts, food broadcasts, and short videos, providing college students with novel and stimulating sensory experiences in addition to taste satisfaction (Huang et al., 2021; Wang, 2020).

### 4.3 Limitations and future directions

This study has limitations. First, the cross-sectional design of this study determines the associations between variables, but it limits the inference of causal relationships between these variables. Second, the data in this study was collected using self-report questionnaires, which may lead to self-report bias. Future research could adopt longitudinal designs. On the one hand, it can examine the causal relationships between handcrafted milk tea consumption, unhealthy lifestyles, and sensation seeking. On the other hand, it can reduce the self-report bias. Thirdly, the study used convenience sampling, with all participants coming from three universities in Chongqing. This limitation restricts the representativeness of the sample; therefore, caution is required when generalizing the findings to college students nationwide. Future research is encouraged to include participants from a wide range of regions and provinces to improve the representativeness and external validity of the results. Fourth, this study did not differentiate among specific types of handcrafted milk tea, instead treating it as a broad concept; subsequent studies could explore the consumption characteristics and determinants of various handcrafted milk tea categories.

## 5 Conclusion

The prevalence of handcrafted milk tea consumption among Chinese college students was 75.2%, with an excessive consumption rate of 7.0%, and handcrafted milk tea consumption was positively associated with unhealthy lifestyles and high sensation seeking; students who consumed carbonated beverages, ate desserts and fried foods, engaged in insufficient physical activity, consumed alcohol, and demonstrated high sensation seeking had an increased prevalence of handcrafted milk tea consumption.

These findings indicate that handcrafted milk tea consumption among college students should not be overlooked, and universities should strengthen education and intervention efforts on handcrafted milk tea consumption from the following three aspects. First, universal education targeting all students should disseminate information on the ingredients of handcrafted milk tea and its potential physical and psychological health risks, thereby enhancing students’ nutrition knowledge and health awareness and promoting more rational consumption of handcrafted milk tea. Second, efforts should be made to strengthen the cultivation of healthy lifestyles by popularizing relevant knowledge and implementing activities such as developing healthy dietary plans, daily physical activity check-ins, and alcohol avoidance, thereby fostering healthy eating and exercise habits and preventing the clustering of unhealthy lifestyles. Third, students should be guided to critically recognize the advertising and marketing strategies of handcrafted milk tea, reduce consumption driven by sensation-seeking motives, and be encouraged to satisfy sensation-seeking needs through positive and socially acceptable healthy risk-taking behaviors, such as travel and physical activity (Hu and Wang, 2019).

## Data Availability

The original contributions presented in the study are included in the article/supplementary material, further inquiries can be directed to the corresponding author.
